# Cold Agglutinin Syndrome and Hemophagocytic Lymphohistiocytosis: An Unusual Combination Caused by Epstein–Barr Virus Infection

**DOI:** 10.7759/cureus.52179

**Published:** 2024-01-12

**Authors:** Beatriz Sousa Nunes, Catarina Gouveia, Paula Kjollerstrom, João Farela Neves

**Affiliations:** 1 Department of Pediatrics, Hospital de Dona Estefânia, Centro Hospitalar Universitário Lisboa Central, Lisbon, PRT; 2 Department of Infectious Diseases, Hospital de Dona Estefânia, Centro Hospitalar Universitário Lisboa Central, Lisbon, PRT; 3 Academic Clinical Centre, Faculdade de Ciências Médicas, Universidade Nova de Lisboa, Lisbon, PRT; 4 Department of Pediatric Hematology, Hospital de Dona Estefânia, Centro Hospitalar Universitário Lisboa Central, Lisbon, PRT; 5 Department of Primary Immunodeficiencies, Hospital de Dona Estefânia, Centro Hospitalar Universitário Lisboa Central, Lisbon, PRT; 6 Comprehensive Health Research Centre, Faculdade de Ciências Médicas, Universidade Nova de Lisboa, Lisbon, PRT

**Keywords:** pediatrics, infectious mononucleosis, hemophagocytic syndrome, epstein–barr virus, autoimmune hemolytic anemia

## Abstract

Autoimmune hemolytic anemia (AIHA) and hemophagocytic lymphohistiocytosis (HLH) are rare complications of infectious mononucleosis. The authors describe a 12-year-old male with acute infectious mononucleosis, hepatitis, cholestasis, and an autoimmune hemolytic disorder caused by cold agglutinins IgM (anti-i specificity). Clinical deterioration with persistent fever, anemia, and hepatosplenomegaly was consistent with cold AIHA plus concomitant HLH. The patient was treated with corticosteroids and acyclovir, with an uneventful recovery. Although rare, cold agglutinin syndrome and HLH can complicate infectious mononucleosis and should be considered in a patient with clinical deterioration. Corticosteroids are the mainstay treatment of HLH and may be beneficial in infection-associated cold agglutinin syndrome.

## Introduction

Epstein-Barr virus (EBV) infection is ubiquitous, with more than 95% of adults worldwide being seropositive [1-2]. Primary infection typically occurs in childhood and infectious mononucleosis (IM) is the main clinical syndrome [3]. Although IM is usually a benign and self-limited disease, rare complications have been described [2,4].

Autoimmune hemolytic anemia (AIHA) is uncommon in children [1,5,6]. It has an estimated incidence of 0.2 per one million individuals younger than 20 years of age, with a peak under the age of five [5]. Cold AIHA represents 25% of all AIHA [6]. In children, it is usually secondary to a concurrent condition, mainly acute infections (EBV, *Mycoplasma pneumoniae*, cytomegalovirus or CMV), autoimmune disorder, or malignancy. This defines an entity called cold agglutinin syndrome (CAS), as opposed to cold agglutinin disease where no underlying clinical disorder is present [6-8].

Hemophagocytic lymphohistiocytosis (HLH) is a rare, life-threatening hyperinflammatory syndrome. In most cases, HLH is triggered by intracellular infections (namely EBV, CMV, mycobacteria, or *Leishmania*), but it can also be associated with malignancies and autoinflammatory syndromes [2,9,10]. Familial HLH (fHLH) is, in most cases, caused by genetic defects of the lymphocyte cytotoxicity, but it can also be related to inborn errors of immunity, inflammation, or even metabolism. Secondary HLH is the term used in cases where a genetic cause is not identified [10]. It is important to consider the possibility of a genetic defect, as early diagnosis and initiation of adequate treatment seem to improve fHLH survival [11].

CAS and HLH are rare entities in children. There are only a few published cases in medical literature of CAS and HLH as complications of a primary EBV infection. We report a case of an unusual association of CAS and HLH in a previously healthy patient with IM.

## Case presentation

A 12-year-old male, with unremarkable previous medical history, was transferred to a tertiary hospital for a febrile illness, pharyngitis, vomiting, and diarrhea for 4 days, with a 48-hour history of acute onset jaundice, abdominal pain, and rash. There was no history of blood product transfusion, recent use of drugs, consanguinity, or family history of early unexpected death, immunodeficiency, or HLH. Physical examination at admission was notable for fever, ill appearance, jaundice, inflamed tonsils, and hepatomegaly (5 cm below the costal margin). 

Laboratory tests revealed a white cell count of 7500 /μL (automated differential: neutrophils 40%; lymphocytes 47%; monocytes 12%), erythrocytes 4.67x106 /μL, hemoglobin 14.2 g/dL, hematocrit 41.2%, reticulocytes 0.95%, and platelet count 128000 /μL. The blood smear showed rare spherocytes and activated lymphocytes. Biochemical testing was notable for total bilirubin of 372.8 mmol/L, direct bilirubin 228.6 mmol/L, aspartate aminotransferase 221 U/L, alanine aminotransferase 197 U/L, γ-glutamyl transferase 193 U/L, alkaline phosphatase 404 U/L, lactate dehydrogenase 1644 U/L, albumin 32.3 g/L, undetectable haptoglobin, C-reactive protein 46.3 mg/L, and erythrocyte sedimentation rate of 50 mm/h (Table 1). 

**Table 1:** Summary of laboratory evolution. ALP: alkaline phosphatase; ALT: alanine aminotransferase; AST: aspartate aminotransferase; CPR: C-reactive protein; ESR: erythrocyte sedimentation rate; GGT: γ-glutamyl transferase; HD: hospital day; LDH: lactate dehydrogenase; MCHC: mean corpuscular hemoglobin concentration; MCV: mean corpuscular volume. Units of measurement are presented in the table for each parameter.

Parameters	Reference range	At admission	3rd HD	6th HD	At discharge	Follow-up 8 weeks
Erythrocytes (cells/μL)	4.5 - 5.3x10^6^	4.7x10^6^	3.15x10^6^	3.51x10^6^	3.7x10^6^	5.3x10^6^
Hemoglobin (g/dL)	13.0 - 16.0	14.2	9.7	10.4	11.6	15.5
Hematocrit (%)	37.0 - 49.0	41.2	27.7	31.0	34.9	46.8
MCV (fL)	78.0 - 96.0	88.2	88.0	88.4	94.1	88.1
MCHC (g/dL)	31.0 - 36.0	34.5	35.0	33.5	33.2	33.1
Reticulocytes (%)	0.50 - 2.50	0.95	1.49	-	-	1.57
Haptoglobin (g/L)	0.30 - 2.00	< 0.07	< 0.07	-	-	1.01
White cell count (cells/μL)	4500 - 13000	7500	11800	15000	13800	7200
Neutrophils (%)	40.0 - 75.0	40.0	64.2	42.1	42.4	42.8
Lymphocytes (%)	15.0 - 45.0	47.0	28.8	47.8	44.1	47.9
Monocytes (%)	2.0 - 11.0	11.9	5.8	9.0	12.4	8.9
Platelets (cells/μL)	150000 - 450000	128000	203000	390000	499000	263000
Total bilirubin (mmol/L)	3.42 - 20.52	372.78	728.80	108.93	68.60	13.20
Direct bilirubin (mmol/L)	0.86 - 4.96	228.63	507.87	77.98	-	-
AST (U/L)	14 - 35	221	193	113	45	19
ALT (U/L)	9 - 25	197	166	201	118	16
GGT (U/L)	7 - 21	193	243	160	115	10
ALP (U/L)	141 - 460	404	410	-	227	297
Triglycerides (mg/dL)	< 150	162	248	331	215	51
Ferritin (ng/mL)	13.7 - 78.8	5483.1	5736.1	2843.0	-	82.9
Fibrinogen (g/L)	1.5 - 4.9	2.4	2.4	1.8	-	-
Albumin (g/L)	38.0 - 54.0	32.3	30.3	-	-	-
LDH (U/L)	157 - 272	1644	-	-	-	191
ESR (mm/h)	< 11	50	63	-	-	6
CPR (mg/L)	< 5.0	46.3	21.6	6.5	9.9	0.8
Immunoglobulin G (g/L)	8.20 - 18.35	11.10	-	-	-	-
Immunoglobulin A (g/L)	0.64 - 4.30	2.01	-	-	-	-
Immunoglobulin M (g/L)	0.70 - 1.50	2.49	-	-	-	-

**Figure 1:**
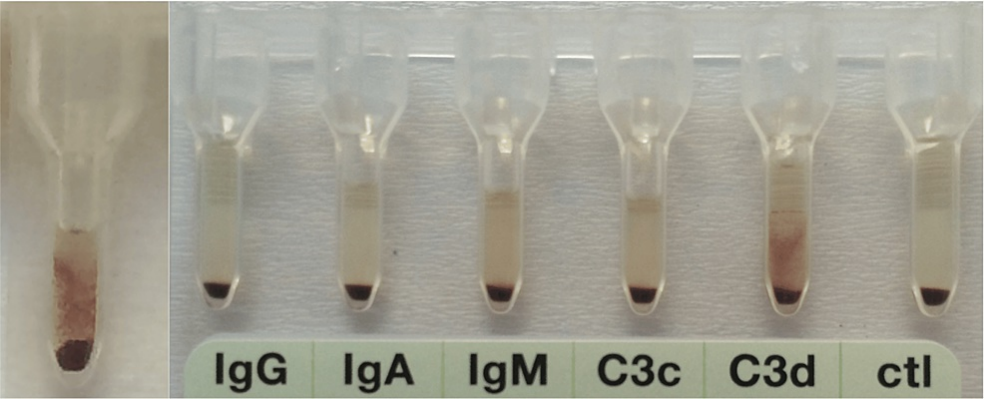
Polyspecific and monospecific direct anti-globulin test. Left: Positive polyspecific direct anti-globulin test. Right: Monospecific direct anti-globulin test positive for C3d and positive for IgM.

Due to evidence of hemolysis, an immunohematological workup was performed. The polyspecific direct anti-globulin test (DAT) was positive, and the monospecific DAT was positive for C3d and IgM (Figure 1). 

Cold agglutinin titration revealed the presence of an IgM auto-antibody with anti-i specificity (titer 1:512). Serum protein electrophoresis showed no gammopathy and the quantitation of immunoglobulins revealed an elevated IgM (Table 1). Serological testing unveiled acute EBV infection (Table 2). 

**Table 2:** Positive serological findings and peripheral blood lymphocyte immunophenotyping. CMV: Cytomegalovirus; EA: Early antigen; EBNA: EBV nuclear antigen; EBV: Epstein–Barr virus; HD: hospital day; PCR: polymerase chain reaction; VCA: viral capsid antigen. *Detection threshold. **At the 3rd hospital day. Units of measurement are presented in the table for each parameter.

Parameters	Reference range	At admission	Follow-up 5 months
EBV - VCA IgG (U/mL)	Positive >25	10	212
EBV - VCA IgM (U/mL)	Positive >40	160	20
EBV - EA IgG (U/mL)	Positive >40	5	132
EBNA - IgG (U/mL)	Positive >20	3	9
PCR EBV whole blood (UI/mL)	*83 - 8x10^6^	**3241.449	Negative
CMV IgG (UA/mL)	Positive >6	214	-
CMV IgM (UA/mL)	Positive >0.9	1	-
PCR CMV urine	-	Negative	-
CD3+ CD8+ T-cells (cells/uL)	330-920	5428	933
CD3+ CD8+ T-cells (%)	18-35	82	37
CD8+HLA DR+ T-cells (cells/uL)	30-180	5145	255
CD8+HLA DR+ T-cells (%)	5-25	95	27
CD19+ B-cells (cells/uL)	110-570	42	285
CD19+ B-cells (%)	6-23	1	11
CD27+IgD- B-cell with switch (cells/uL)	12-69	1	9
CD27+IgD- B-cell with switch (%)	4-21	3	3
Soluble CD25 (pg/mL)	458-1997	7996	896

An abdominal Doppler ultrasonogram showed a homogenous hepatosplenomegaly (liver length of 18.1 cm and spleen length of 15.4 cm), a distended gallbladder and biliary sludge without gallstones or dilatation of the biliary tract, and no signs of hepatic vascular thrombosis. A diagnosis of an acute EBV infection (Table 2), associated with hepatitis, cholestasis, and compensated cold agglutinin-mediated autoimmune hemolysis was evoked. He was initially started on ursodeoxycholic acid (10 mg/kg/day divided into two doses).

**Figure 2:**
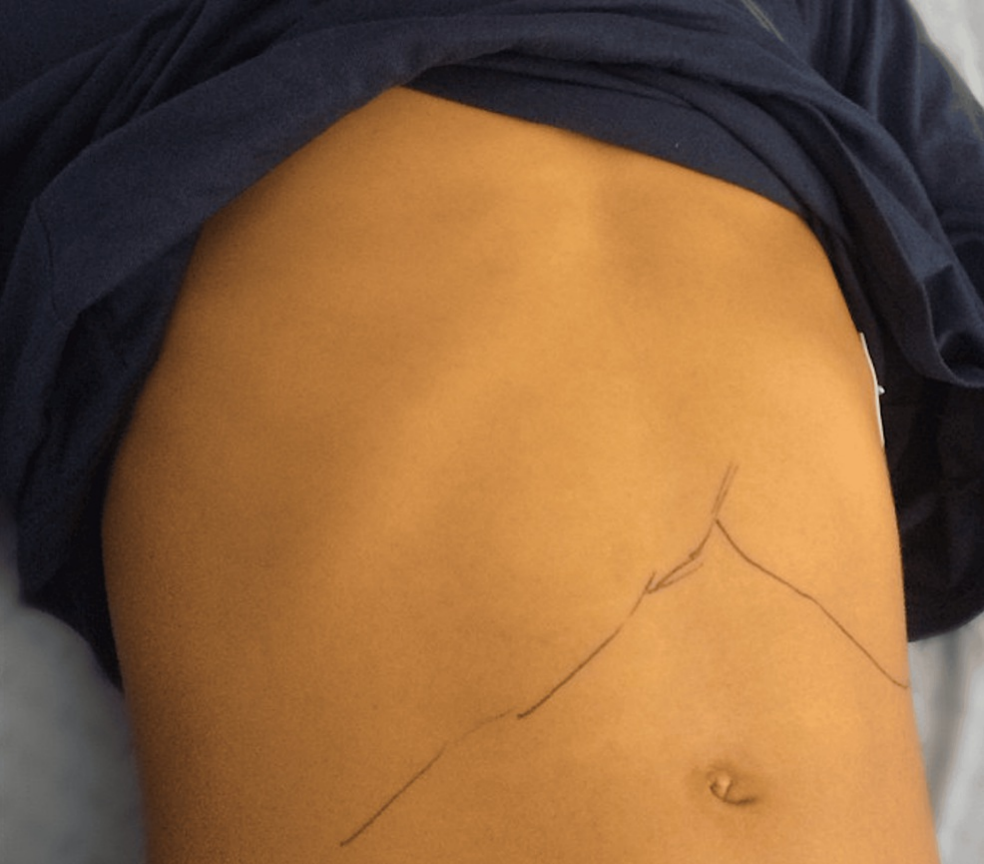
Adolescent male with infectious mononucleosis caused by an acute Epstein–Barr virus infection, complicated with hepatitis, cholestasis, cold agglutinin-mediated autoimmune anemia, and hemophagocytic lymphohistiocytosis. Picture taken on the 3rd hospital day (day 7 of illness). Note the jaundice and the drawn limits of the liver and spleen margins.

On the 7th day of illness, the patient remained feverish with worsening of the jaundice and hepatosplenomegaly (Figure 2).

The serial blood workup was notable for hemoglobin 9.7 g/dL, reticulocytes 1.49%, total bilirubin 728.8 mmol/L, direct bilirubin 507.9 mmol/L, fasting triglycerides 248 mg/dL, ferritin 5736.1 ng/mL, as well as elevated soluble CD25, thus fulfilling six HLH criteria (Table 1). Interestingly, the peripheral blood lymphocyte immunophenotyping revealed a marked elevation of activated CD8+ T-cells (95% of the CD8+ were HLA DR+), and the CD8 VBeta repertoire revealed the absence of oligoclonality.

The diagnosis of EBV‐associated HLH (EBV-HLH) and cold-agglutinin-mediated hemolytic anemia were made. He was started on acyclovir and dexamethasone (10 mg/m2/day). There was a dramatic response within 3 days, becoming afebrile, with normalization of hemoglobin, bilirubin, and inflammatory markers (Table 1). The patient was discharged on the 9th hospital day. Dexamethasone was switched to prednisolone, which was tapered in the next 3 weeks. 

After 8 weeks, the physical examination was unremarkable and all laboratory data was normal. The 5-month follow-up excluded chronic active EBV with negative EBV viremia and EBV antibody seroconversion (Table 2). Unfortunately, he was then lost to follow-up and a genetic study (Whole Exome Sequencing-based 477 Gene Primary Immunodeficiency Panel) was not pursued.

## Discussion

We describe a case of an acute EBV infection, with hepatitis, cholestasis, and cold agglutinin-mediated autoimmune hemolysis. The clinical picture was complicated by the unusual association of CAS and HLH.

IM is the main clinical syndrome of EBV infection, and it is characterized by fever, pharyngitis, benign lymphoproliferation, malaise, and atypical lymphocytes [3]. Our patient had all the classic signs and symptoms of IM. Although IM is usually benign and self-limited, rare complications can include hepatitis, thrombocytopenia, hemolytic anemia, aplastic anemia, neutropenia, splenic rupture, HLH, autoimmunity, and malignancy [2,4].

Cold agglutinins develop in more than 60% of patients with IM; nonetheless, AIHA is rare [3-4]. EBV-associated AIHA occurs in around 0.5-3% of hospitalized patients with IM [4,12], although the majority of the IM patients are not hospitalized, so the true prevalence is likely much lower than 1% [12].

Our patient was admitted with a compensated hemolytic process, with elevated serum levels of lactate dehydrogenase, unconjugated bilirubin, and low levels of haptoglobin. The features of the blood smear showed rare spherocytes without other morphologic changes in the red lineage. These findings suggested intra and extravascular hemolysis. In the first week of disease, the patient became anemic. Although the EBV-associated AIHA usually manifests in the first 2 or 3 weeks of disease [4], it has been reported as less commonly occurring simultaneously with IM [13]. The development of HLH can also explain this earlier onset of the anemia. 

The monospecific DAT was positive for C3d and IgM. The initial anti-IgM weak positive DAT result was confirmed by a tube test. IgM is a potent complement activator and its activation leads to the coating of erythrocytes with C3b. C3b-opsonized cells are phagocytosed by the mononuclear system, primarily by Kupffer cells of the liver (extravascular hemolysis) [6-7]. Less often, C3b can also react to form C5 convertase and initiate the terminal complement leading to the formation of the membrane attack complex (intravascular hemolysis) [6-7]. To assess the clinical significance of this antibody, the cold agglutinin titer was assessed. When the cold agglutinin titer is at least 64, it is considered abnormal [6-7]. Our patient had a high titer of 152 with anti-i specificity. The thermal amplitude of the antibody was not assessed, but this testing is usually not a regular step of the evaluation of cold agglutinins, nor it is necessary for the diagnosis of cold AIHA [6]. In CAS secondary to infections, the immune response is usually polyclonal. The target of autoantibodies produced after EBV infection is the i blood group antigen [6,8].

The overt hemolysis, the presence of high-titer cold antibodies, the positive DAT with anti-i specificity together with a detectable viral load for EBV support the diagnosis of cold AIHA due to EBV infection.

EBV-HLH can vary from mild to severe [14]. The distinction between primary EBV infection and HLH is sometimes difficult, as they both share some hallmark characteristics and no firm clinical diagnostic thresholds are defined [15]. The suspicion of HLH in our patient was derived from prolonged fever, splenomegaly, hypertriglyceridemia, hyperferritinemia, and elevated soluble CD25. The anemia was attributed to AHAI, and no other cytopenia was noted. No coagulopathy, cutaneous, or neurological symptoms were recognized. Although EBV is the most common infectious trigger, HLH is rare in children with IM. In a retrospective study of 513 pediatric patients with primary EBV infection, only three (0.6%) patients developed EBV‐associated HLH (EBV-HLH) and only two had concomitant IM [16]. To our knowledge, the prevalence of CAS in patients with HLH has not been reported. Interestingly, a patient with primary HLH and IgG cold agglutinins, without evidence of hemolysis has been previously described [17].

Our patient’s hyperbilirubinemia resulted from increased indirect bilirubin from AIHA along with increased concentrations of direct bilirubin. The co-existing obstructive pattern with abnormal hepatic panel has been reported in other case reports of EBV infection complicated by hemolytic anemia [4,12,18]. This can also be explained by hepatocyte damage secondary to EBV infection/HLH leading to a reduction in the conjugation and excretory capacity of the liver, reduction of bile flow, or obstruction of the lymphatic flow secondary to enlarged lymph node [4].

There are no randomized controlled studies regarding therapeutic recommendations of AIHA in pediatric patients, and no systematic study has been published on CAS [5,6,8]. The treatment of CAS is directed at the underlying disease. The hemolytic anemia is self-limited [6-8] and usually resolves in 2 weeks in EBV-associated AIHA [4]. Transfusions are safe, regarding that the extremity should be kept warm and a blood warmer should be used during infusion [6-8]. The use of corticosteroids [8] and complement inhibition [7-8] in infection-associated CAS has been described in small reports, but no good evidence of benefit has been published. A stepwise approach to the treatment of EBV-HLH has been widely discussed [14]. Our patient presented with a mild EBV-HLH, with relatively low viremia and no organ failure, leading to the adoption of a conservative approach. He was started on acyclovir and intravenous dexamethasone, followed by prednisolone for a total of 3 weeks. Due to the clinical improvement and uneventful recovery, we decided not to start rituximab. Since the corticosteroid therapy is associated with substantially lower EBV viral loads and has shown to be beneficial in some immunocompetent patients with complicated IM [12], we decided to continue both prednisolone and acyclovir for 3 weeks, when the EBV viral load became negative. The 5-month follow-up revealed a complete resolution of the clinical picture and excluded chronic active EBV.

## Conclusions

We report rare case of CAS and HLH in a previously healthy recipient with IM, treated conservatively. Multiple considerations should be taken into account in the clinical practice. Although rare, CAS and HLH can complicate IM. A high suspicion index of AIHA is necessary when a patient presents with EBV infection associated with hepatitis/cholestasis, with conjugated hyperbilirubinemia rather than unconjugated, and an immunohematological workup should be performed. A positive DAT for C3d±IgM with anti-i specificity in the context of acute EBV infection supports the diagnosis, even without testing the thermal amplitude of the antibody. HLH should be consider in the context of IM with clinical deterioration. The best therapeutical approach has not been yet defined. Corticosteroids are the mainstay treatment of HLH and may be beneficial in infection-associated CAS.
